# Secondary infertility due to intrauterine fetal bone retention: A case report and review of the literature

**DOI:** 10.18502/ijrm.v17i8.4825

**Published:** 2019-09-03

**Authors:** Atossa Mahdavi, Sasan Kazemian, Emad Koohestani

**Affiliations:** ^1^ Department of Obstetrics, Gynecology and Infertility, Shariati Hospital, Tehran University of Medical Sciences Tehran Iran.; ^2^ Shahed University Tehran Iran.; ^3^ Tehran University of Medical Science Tehran Iran.

**Keywords:** Bone, Infertility, Hysteroscopy, Pregnancy, Abortion

## Abstract

**Background:**

Intrauterine retention of fetal bone fragments is a rare condition that could happen after abortion (especially illegal abortion). It can cause secondary infertility as bone fragments can work as an intrauterine contraceptive device.

**Case:**

A 25-year-old Iranian woman was referred to Shariati Hospital due to infertility. During infertility work up to normal semen analysis, adequate ovarian reserve with regular ovulatory cycles was documented. An ultrasound scan revealed focal echogenic shadowing lesions inside the uterine cavity. Hysteroscopy was conducted and many intrauterine bone fragments were revealed. Six months after hysteroscopic removal of fetal bones, the patient became pregnant and delivered a healthy and term baby.

**Conclusion:**

Intrauterine fetal bone retention is a scarce event that happens after pregnancy termination due to the incomplete evacuation of fetal tissues. It can cause dysfunctional uterine bleeding, menorrhagia, dysmenorrhea, pelvic pain, abnormal vaginal discharge, and secondary infertility. The detection of the problem and the removal of the remained bones by hysteroscopy have made possible to treat the patient safely and restore normal uterine function and female fertility.

## 1 Introduction

Retention of fetal bone fragments in the uterus is a rare condition that usually happens after pregnancy termination in the second or third trimesters of pregnancy (1, 2). Retained fetal fragments may cause pelvic pain, vaginal discharge, abnormal uterine bleeding, and secondary infertility. The true incidence is not known, but it is important to be recognized and managed as soon as possible because it is a potentially treatable cause of secondary infertility. These bony fragments work as an intrauterine contraceptive device (IUCD) and stimulate prostaglandin secretion by endometrial tissue causing secondary infertility (3, 4). Hysteroscopy as a diagnostic and curative method to remove these bony parts can restore fertility in most of these patients (1–5).

In this case report, we present a patient with secondary infertility who did reveal her previous illegal abortion only after hysteroscopy removal of the pieces of fetal bone.

## 2 Case presentation

A 25-yr-old Iranian infertile woman was referred to the Shariati Hospital affiliated to the Tehran University of Medical Sciences. She was a healthy young lady with the problem of 1.5 yr of infertility. She mentioned regular menstrual cycles with normal volume and duration of bleeding without any pain during or between the menses. She didn’t report any history of abortion or even pregnancy before in her first visit. A routine gynecological examination didn’t reveal any pathologic finding. Semen analysis of her husband was normal. Her lab tests and hysterosalpingography were normal. An ultrasound scan revealed focal echogenic shadowing lesions inside the uterine cavity.

**Figure 1:**
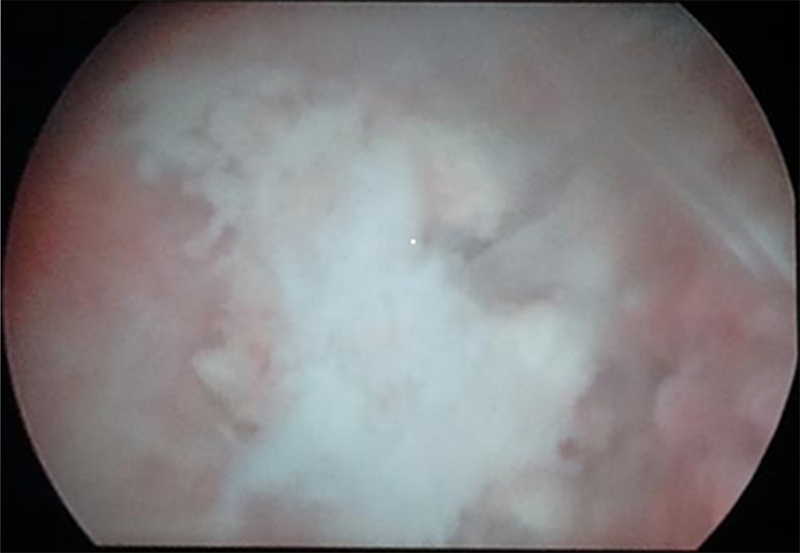
Retained fetal bone in the uterus.

**Figure 2:**
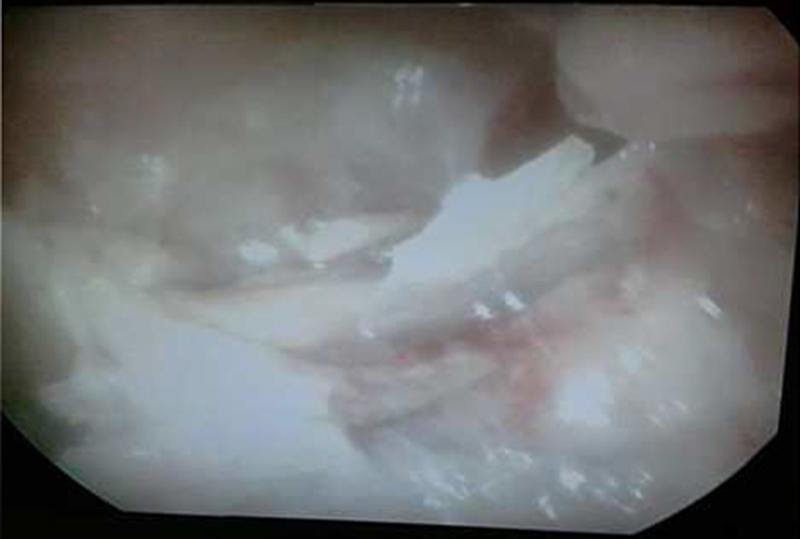
Multiple retained fetal bones in the uterus.

Hysteroscopy under general anesthesia revealed that her uterus was filled with many intrauterine bone fragments with clear fetal features (Figures 1, 2).

Then, fetal bone pieces of 2–3░cm were removed by hysteroscopy. Samples were sent for pathology studies that show fetal bones and chronic inflammation.

After the procedure, the patient confessed that she had an illegal abortion 2 yr ago at the gestational age of 15 wk. The patient was reminded that the previous abortion was not a complete pregnancy termination and this fetal remnant worked as an intrauterine device that prevented pregnancy occurrence. After six months, she reported her positive pregnancy test and after nine months she delivered a healthy newborn.

### 2.1 Ethical consideration

Written informed consent was obtained from patient.

## 3 Discussion

Intrauterine fetal bone retention is a scarce event. It happens after an induced pregnancy termination especially during second and third trimesters due to the incomplete evacuation of fetal tissues (1, 2, 6). Bone fragments cause some symptoms such as dysfunctional uterine bleeding, menorrhagia, dysmenorrhea, pelvic pain, abnormal vaginal discharge, and infertility (7). Although, according to genetic analysis, bone formation caused by osseous metaplasia due to inflammation, has been reported in eight women with histories of previous abortion (8). This bone formation can interfere with normal uterine function and can work as a contraceptive resulting in secondary infertility or abortion. There are recent reports of calcified tissue of fetal origin in utero (9), with the oldest article about the exogenous foreign body in uterus returning back to 1966 (10). In a systematic review published in 2016, infertility with a frequency of 56% was the most common presenting symptom and only 5% of the patients were asymptomatic (11). Irregular bleeding, pain symptoms, and infection were reported in 20%, 12%, and 6.5% of patients, respectively. Interestingly, the interval between the last pregnancy and the time of presentation or incidental diagnosis ranged from 1 to 40 (median of 5░yr) (11).

In this case, fetal bones remained from previous abortion that was performed 24 months ago. As we know any external object may cause gynecological symptoms such as dysmenorrhea, menorrhagia, pelvic pain, vaginal discharge, but in this case despite bone retention there was not any gynecologic complaint. The patient was only suffering from secondary infertility despite being a potentially fertile woman. The patient’s denial about previous elective abortion because of legal and religious issues did not reveal the reason for infertility during the work up. It was revealed only after hysteroscopy as a valuable tool to diagnose and manage the patient safely. This case highlights the importance of precise history-taking during patient visits, especially in our crowded public clinics. Also, this case elucidates the importance of awareness and vigilance about illegal abortions especially in countries where abortion is not legally permitted. However, in this case, hysteroscopy was a valuable tool to manage the patient safely. The removal of the remained bones restored fertility. Recent progressions in hysteroscopy technique have made it possible to detect and remove bones easily and with least side effects.

An interesting finding from this report is the ability to become pregnant soon after the operation. Our patient became pregnant six months after hysteroscopy and immediately after her decision to become pregnant. It is in accordance with Khan *et al.*’s finding that spontaneous pregnancy or symptom relief were remarkable (11). But they reminded about the possibility of increased spontaneous miscarriage due to the remnants of osseous material in the endometrium. Fortunately, our patient delivered a healthy term baby.

Medical abortion is not a legal procedure in most Middle Eastern countries including Iran. Some women with an unwanted pregnancy are forced to perform it in the poor hygienic setting (known as backstreet abortion) by inexperienced or non-qualified people, sometimes even by the mother herself (called self-induced). Also, later pregnancy termination in these regions increases the probability of incomplete evacuation, resulting in more mortality and morbidity complications. Increased public awareness besides revisions in some rules can help to prevent the morbidities and mortalities. Moreover, consideration of available, cheap, and effective contraceptive methods and sexual health education should be warranted in every community.

This case report highlights the role of hysteroscopy in the diagnosis and treatment of secondary infertility caused by fetal bone retention; so we recommend this method as the first step in the evaluation of the same cases. However, ultrasound scan reserves its place for the primary diagnosis, to raise the clinician suspicion for pathologies that interfere with fertility. We also remind to remove all fetal tissues after an abortion to avoid fetal bone retention and future fertility problems.

## Conflict of Interest

There was no conflict of interest.
